# Associations of age, comorbidities, and inflammatory markers with disease severity in pediatric human metapneumovirus infection

**DOI:** 10.1007/s00431-026-06936-0

**Published:** 2026-04-16

**Authors:** Kübra Nur Erdoğan, Mustafa Gençeli, Esra Babayiğit, Ayşe Şimşek, Talha Üstüntaş, Özge Metin Akcan, Fatih Ercan, Sipil Gençeli, Mehtap Yücel, Abdullah Akkuş, Sevgi Pekcan

**Affiliations:** 1 Faculty of Medicine, Departments of Pediatrics, Necmettin Erbakan University, Konya, Turkey; 2 Faculty of Medicine, Departments of Pediatric Infectious Diseases, Necmettin Erbakan University, Konya, Turkey; 3 Faculty of Medicine, Department of Pediatric Pulmonology, Necmettin Erbakan University, Konya, Turkey; 4 Faculty of Medicine, Department of Public Health, Necmettin Erbakan University, Konya, Turkey

**Keywords:** Human metapneumovirus, Disease severity, Comorbidity, Inflammatory markers, Lower respiratory tract infection, Pediatrics

## Abstract

To examine factors associated with severe respiratory disease at initial presentation in pediatric hMPV-associated LRTIs. We analyzed patients aged 1 month to 18 years with PCR-confirmed hMPV-associated LRTI (January 2018–January 2024). Clinical severity was stratified via the Modified Tal Score at admission (mild ≤ 5, moderate 6–10, severe ≥ 11). Each patient contributed one episode. Multivariable binary logistic regression identified risk factors for severe respiratory disease; ROC analysis evaluated biomarker diagnostic accuracy. A total of 676 hMPV-positive patients were identified; after restricting to one episode per patient, 421 unique episodes were analyzed. Of these, 54.9% were mild, 38.5% moderate, and 6.7% (*n* = 28) severe. Comorbidities were present in 67.9% of severe versus 35.5% of mild cases (*p* = .004). Viral co-detections occurred in 41.3% but were unrelated to severity (*p* = .235). Median CRP was 4.0, 10.0, and 32.0 mg/L in mild, moderate, and severe groups, respectively (p < .001). In multivariable regression, comorbidity was the strongest risk factor (OR 2.96, 95% CI 1.17–7.49, *p* = 0.022), followed by CRP (OR 1.55 per 1 SD [≈32 mg/L], *p* = 0.018). No age group retained significance after adjustment (model AUC 0.733). *Conclusions*: Severe respiratory disease was infrequent (6.7%) and associated primarily with underlying comorbidities, which increased the odds approximately threefold. Age was not independently associated with severity. CRP showed modest diagnostic accuracy (AUC 0.678, negative likelihood ratio 0.59); low levels alone could not reliably exclude severe respiratory disease.

**Table:** 

**What is Known:** • *hMPV is an established cause of lower respiratory tract infections in children, Ranging from mild illness to severe pneumonia requiring hospitalization and respiratory support.* • *Infants younger than 12 months and children with comorbidities such as prematurity, chronic lung disease, congenital heart disease, or neurological disorders carry increased risk for severe hMPV infection.*
**What is New:** • *Multivariable logistic regression identifies underlying comorbidity as the dominant independent risk factor for severe hMPV respiratory disease (OR 2.96, p = 0.022). No age group retains significance after adjustment, indicating that the apparent severity signal in children older than 5 years reflects a comorbidity-driven population shift rather than older age acting as an independent risk factor.* • *Elevated CRP, decreased lymphocyte counts, and decreased monocyte counts at admission are associated with severe respiratory disease. CRP retained independent association in multivariable analysis, though diagnostic accuracy for all three biomarkers was modest.*

**What is Known:**

• *hMPV is an established cause of lower respiratory tract infections in children, Ranging from mild illness to severe pneumonia requiring hospitalization and respiratory support.*

• *Infants younger than 12 months and children with comorbidities such as prematurity, chronic lung disease, congenital heart disease, or neurological disorders carry increased risk for severe hMPV infection.*

**What is New:**

• *Multivariable logistic regression identifies underlying comorbidity as the dominant independent risk factor for severe hMPV respiratory disease (OR 2.96, p = 0.022). No age group retains significance after adjustment, indicating that the apparent severity signal in children older than 5 years reflects a comorbidity-driven population shift rather than older age acting as an independent risk factor.*

• *Elevated CRP, decreased lymphocyte counts, and decreased monocyte counts at admission are associated with severe respiratory disease. CRP retained independent association in multivariable analysis, though diagnostic accuracy for all three biomarkers was modest.*

## Introduction

Human metapneumovirus (hMPV) is a significant etiology of respiratory tract infections worldwide, particularly within the pediatric population [1]. In regions with temperate climates, viral activity often peaks during the late winter and early spring months, which coincides temporally with the circulation patterns of respiratory syncytial virus (RSV) and influenza [2].

The clinical spectrum documented in pediatric patients mirrors that of RSV disease and encompasses manifestations ranging from mild upper respiratory illness to life-threatening bronchiolitis and pneumonia. Infants and young children affected by hMPV may require hospitalization and respiratory support via mechanical ventilation [2, 3]. Among children under age five, severe clinical courses are predominantly reported in patients with underlying comorbidities, including prematurity, chronic pulmonary disease, structural cardiac abnormalities, neuromuscular disorders, immunocompromised states, and trisomy 21 [4]. Concurrent infections involving additional respiratory pathogens are frequently identified, highlighting the prevalence of polymicrobial respiratory illness in this demographic [5].

To stratify clinical severity, physicians often employ validated instruments such as the Modified Tal Score (M-Tal), which assesses respiratory rate, wheezing, oxygen saturation, and accessory muscle recruitment to inform therapeutic interventions and admission decisions [6]. Although hMPV infection typically manifests as a self-limiting, mild respiratory illness, disease progression to severe forms can occur, which may result in fatal outcomes even among previously healthy children [7]. We aimed to identify the demographic, clinical, and laboratory factors associated with the degree of respiratory compromise at initial presentation in pediatric patients with hMPV-associated LRTI, as quantified by the Modified Tal Score at admission. Because viral panel results were not available at the point of care, we focused on clinical and laboratory parameters measurable at admission that could support early risk stratification.

## Methods

This retrospective observational cohort study was conducted at the Pediatric Emergency Department, inpatient pediatric wards, and outpatient clinics of a tertiary-care university hospital that serves as a regional referral center. The hospital accepts both direct admissions and referrals from primary and secondary healthcare facilities. The study period spanned 01 January 2018 to 31 January 2024.

Study population comprised patients aged 1 month to 18 years presenting with a diagnosis of LRTI and PCR-confirmed hMPV from nasopharyngeal swabs during the study period. Infants under 1 month were excluded to prevent diagnostic heterogeneity, as they follow separate neonatology protocols. Data were retrieved from electronic medical records.

Respiratory viral testing was performed routinely in all patients diagnosed with LRTI according to our institutional protocol, regardless of perceived severity. Samples were collected by physicians within the first 4 h of presentation, before treatment initiation and before hospitalization decisions for outpatients. Viral respiratory pathogens were detected via a multiplex real-time RT-PCR respiratory panel (Bioeksen R&D Technologies, Istanbul, Türkiye). Because panel results took ≥ 72 h, patient management and hospitalization decisions relied entirely on clinical evaluation rather than viral etiology. No study-specific diagnostic or therapeutic protocol was implemented; patient management reflected routine clinical practice throughout.

Initial data extraction identified 676 hMPV-positive records from the hospital registry. Because individual patients could appear more than once in the registry across the 6-year study period, each patient was restricted to a single index episode to maintain statistical independence. All subsequent analyses were performed on the resulting 421 unique patient episodes. Disease severity was retrospectively stratified at admission into mild (≤ 5), moderate (6–10), and severe (≥ 11) categories using the Modified Tal Score (Table 1), which sums four component scores (respiratory rate, oxygen saturation, wheezing, and accessory muscle use) to yield a total range of 0–12. This retrospective scoring was independent of real-time admission decisions. We used this score rather than hospitalization status for severity classification to minimize subjective clinical admission biases. LRTI was defined as an acute respiratory illness with cough and/or dyspnea accompanied by at least one lower respiratory sign on physical examination (wheezing, crackles, decreased breath sounds, or respiratory distress signs such as nasal flaring, grunting, or intercostal/subcostal retractions) and/or radiological evidence of bronchiolitis or pneumonia on chest X-ray. Children who presented with isolated upper respiratory symptoms like rhinorrhea, nasal congestion, sore throat without auscultatory findings or radiologic abnormalities were classified as upper respiratory tract infection and excluded.

**Table 1:** Modified Tal score assessment criteria

Score	RR (Age < 6 mo)	RR (Age ≥ 6 mo)	SpO₂ (%)	Wheezing/Fine Crepitations	Accessory Muscle Use
0	≤ 40	≤ 30	≥ 95	None	None
1	41–55	31–45	92–94	Expiration only	Mild (+)
2	56–70	46–60	90–91	Expiration and inspiration with stethoscope only	Moderate (+ +)
3	≥ 71	≥ 61	≤ 89	Expiration and inspiration without stethoscope	Severe (+ + +)

SpO₂ = peripheral oxygen saturation. RR = respiratory rate (breaths per minute). The total score is the sum of all four component scores (range 0–12). Clinical severity: Mild (≤ 5), Moderate (6–10), Severe (≥ 11)

Analyses were performed in SPSS for Windows, version 18.0 (SPSS Inc., Chicago, IL, USA). Non-normally distributed continuous variables are reported as medians (interquartile ranges) and categorical variables as frequencies (percentages). Group comparisons used the Chi-square test, Kruskal–Wallis test (with Dunn-Bonferroni post-hoc analysis), or Mann–Whitney U test, as appropriate. Significance was set at *p* < 0.05.

Multivariable binary logistic regression identified independent risk factors for severe respiratory disease (Modified Tal Score ≥ 11), reporting odds ratios (ORs) and 95% confidence intervals (CIs). The model evaluated age group, comorbidity, and standardized continuous biomarkers (CRP, NLR, monocyte count). Given 27 severe events, the model was restricted to six predictors. Patients with incomplete laboratory data for any of the selected biomarkers were excluded, which yielded a final regression sample of 322 unique episodes. Receiver operating characteristic (ROC) curve analysis was performed for each biomarker that showed significant univariate differences, with optimal cut-off values determined by Youden's Index. Sensitivity, specificity, positive predictive value (PPV), negative predictive value (NPV), and negative likelihood ratio were calculated at each threshold, and AUC values are reported with 95% CIs.

The study was approved by the Ethics Committee of Necmettin Erbakan University Faculty of Medicine (Decision No. 2025/5674) and all study protocols adhered to the principles of the Declaration of Helsinki. The committee waived the requirement for informed consent given the retrospective design.

## Results

During the study period, 676 hMPV-positive records were extracted from the hospital registry. Application of the one-episode-per-patient restriction yielded a final analytic cohort of 421 unique patient episodes. Of these, 54.9% (*n* = 231) were classified as mild, 38.5% (*n* = 162) as moderate, and 6.7% (*n* = 28) as severe. Clinical severity differed significantly across age groups (*p* < 0.001). Moderate cases clustered predominantly in the 1–5-year age bracket, while severe cases were more frequent in both infants (aged < 1 year) and children older than 5 years.

**Fig. 1:**
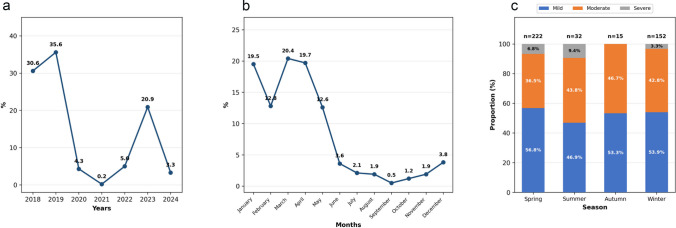
Temporal distribution of hMPV-positive patients. **a** Annual distribution of cases between 2018 and 2024, shown as proportions of the study cohort. **b** Monthly distribution of cases. **c** Seasonal distribution by defined meteorological season with disease severity breakdown

The proportion of hMPV cases fluctuated across the study period. As illustrated in Fig. 1a, the largest proportions were recorded in the pre-pandemic years (30.6% in 2018 and 35.6% in 2019), followed by a sharp decline to 0.2% in 2021 during the height of the COVID-19 pandemic. A gradual resurgence followed, with the cohort proportion rising to 20.9% by 2023. Monthly distribution (Fig. 1b) showed a periodicity with peaks during late winter and spring. The highest proportions were recorded in March (20.4%), April (19.7%), and January (19.5%), while activity was minimal during summer and early autumn, with a nadir in September (0.5%).

Seasonal analysis by defined meteorological seasons confirmed that hMPV activity was concentrated in spring (March–May: *n* = 222, 52.7%) and winter (December–February: *n* = 152, 36.1%), which together accounted for 88.8% of all cases. Summer (June–August: *n* = 32, 7.6%) and autumn (September–November: *n* = 15, 3.6%) showed minimal activity. Severity distributions within spring and winter varied, though not significantly (Fig. 1c). The association between clinical severity and seasonality did not reach statistical significance (*p* = 0.198).

Concurrent viral pathogens were identified in 41.3% (*n* = 174) of the cohort but were not significantly associated with clinical severity (*p* = 0.235). Underlying comorbidities were present in 39.4% (*n* = 166) of the total cohort, and 67.9% of patients with severe respiratory disease had at least one chronic condition. The proportion of patients with comorbidities increased across severity groups (*p* = 0.004). Among specific conditions, neurological disorders, asthma, and Down syndrome were each associated with severity (*p* = 0.001, *p* = 0.048, and *p* = 0.040, respectively). Other chronic disease categories did not differ significantly across groups. Among children older than 5 years, 54.1% had at least one underlying comorbidity. Table 2 presents the distribution of gender, age, seasonality, co-detections, and comorbidities stratified by clinical severity.

**Table 2:** Distribution of demographic and epidemiological characteristics according to clinical severity

Variable	All (*n* = 421)	Mild (*n* = 231)	Moderate (*n* = 162)	Severe (*n* = 28)	*p*
**Gender**					.883
Female	176 (41.8)	95 (41.1)	70 (43.2)	11 (39.3)	
Male	245 (58.2)	136 (58.9)	92 (56.8)	17 (60.7)	
**Age Groups**					<.001
< 1 year	119 (28.3)	57 (24.7)	55 (34.0)	7 (25.0)	
1–5 years	193 (45.8)	100 (43.3)	84 (51.9)	9 (32.1)	
> 5 years	109 (25.9)	74 (32.0)	23 (14.2)	12 (42.9)	
**Season**					.198
Spring	222 (52.7)	126 (54.5)	76 (46.9)	20 (71.4)	
Summer	32 (7.6)	15 (6.5)	14 (8.6)	3 (10.7)	
Autumn	15 (3.6)	8 (3.5)	7 (4.3)	—	
Winter	152 (36.1)	82 (35.5)	65 (40.1)	5 (17.9)	
**Viral Co-detection**	174 (41.3)	104 (45.0)	60 (37.0)	10 (35.7)	.235
Rhinovirus	96 (55.2)	63 (60.6)	29 (48.3)	4 (40.0)	.193
Parainfluenza	16 (9.2)	9 (8.7)	6 (10.0)	1 (10.0)	.956
Enterovirus	8 (4.6)	5 (4.8)	3 (5.0)	—	—
RSV	35 (20.1)	17 (16.3)	16 (26.7)	2 (20.0)	.283
Influenza	13 (7.5)	7 (6.7)	4 (6.7)	2 (20.0)	.300
Coronavirus	11 (6.3)	5 (4.8)	6 (10.0)	—	—
Bocavirus	10 (5.7)	5 (4.8)	5 (8.3)	—	—
Adenovirus	20 (11.5)	17 (16.3)	2 (3.3)	1 (10.0)	.042
**Underlying Comorbidity**	166 (39.4)	82 (35.5)	65 (40.1)	19 (67.9)	.004
Asthma	39 (23.5)	26 (31.7)	10 (15.4)	3 (15.8)	.048
Immunodeficiency	39 (23.5)	20 (24.4)	14 (21.5)	5 (26.3)	.878
Congenital heart disease	21 (12.7)	7 (8.5)	12 (18.5)	2 (10.5)	.190
Down syndrome	9 (5.4)	1 (1.2)	7 (10.8)	1 (5.3)	.040
Hematologic disease	11 (6.6)	4 (4.9)	4 (6.2)	3 (15.8)	.222
Neurologic disease	25 (15.1)	4 (4.9)	16 (24.6)	5 (26.3)	.001
Chronic lung disease	36 (21.7)	20 (24.4)	13 (20.0)	3 (15.8)	.653
Cystic fibrosis	17 (10.2)	11 (13.4)	3 (4.6)	3 (15.8)	.152
Other diseases	19 (11.4)	7 (8.5)	11 (16.9)	1 (5.3)	.190

Values are presented as n (%). p values were calculated via the Chi-square test. Dash (—) indicates insufficient data for statistical calculation or zero value. Viral co-detection percentages are among the 174 co-detected patients. Comorbidity subcategory percentages are among the 166 patients with at least one underlying comorbidity

Hospital admission was required in 47.0% (*n* = 198) of the cohort. Among severe cases, 10.7% (*n* = 3) required intensive care unit admission. Respiratory support requirements increased significantly with increasing severity (Table 3). Median hospital stay was significantly longer for severe cases (14.0 days) compared to mild (8.0 days) and moderate (5.0 days) cases (*p* < 0.001).

**Table 3:** Distribution of hospitalization characteristics and respiratory support requirements according to clinical severity

Variable	All (*N* = 421)	Mild (*n* = 231)	Moderate (*n* = 162)	Severe (*n* = 28)
**Hospitalization**				
Absent	223 (53.0)	223 (96.5)	—	—
Present	198 (47.0)	8 (3.5)	162 (100.0)	28 (100.0)
**ICU Admission**	3 (0.7)	—	—	3 (10.7)
**Respiratory Support**				
None	268 (63.7)	227 (98.3)	38 (23.5)	3 (10.7)
Oxygen	76 (18.1)	3 (1.3)	67 (41.4)	6 (21.4)
High-flow nasal cannula	66 (15.7)	1 (0.4)	56 (34.6)	9 (32.1)
Bilevel Positive Airway Pressure	8 (1.9)	—	1 (0.6)	7 (25.0)
Mechanical ventilation	3 (0.7)	—	—	3 (10.7)
**Mortality**	1 (0.2)	—	—	1 (3.6)

Values are presented as n (%). Dash (—) indicates zero value

Laboratory parameters differed significantly across severity groups for lymphocyte counts, monocyte counts, CRP levels, and NLR (*p* = 0.015, *p* = 0.025, *p* < 0.001, and *p* = 0.021, respectively). Lymphocyte and monocyte levels were lower in the severe group than in the mild group. Median CRP levels were 4.0 (IQR 0.0–17.5) mg/L in the mild group, 10.0 (2.0–30.8) mg/L in the moderate group, and 32.0 (6.5–52.0) mg/L in the severe group; CRP was significantly higher in both the severe and moderate groups relative to the mild group (Severe > Mild, *p* < 0.001; Moderate > Mild, *p* = 0.001), though the difference between severe and moderate groups did not reach statistical significance after Bonferroni correction. NLR was elevated in the severe group relative to the mild group. Leukocyte counts, neutrophil counts, and the monocyte-to-lymphocyte ratio (MLR) did not differ significantly across groups (Table 4).

**Table 4:** Comparison of laboratory parameters across clinical severity groups

Variable	All (*n* = 421)	Mild (*n* = 231)	Moderate (*n* = 162)	Severe (*n* = 28)	*p*
Leukocyte (/mm^3^)	9000 (6520–11750)	9170 (6975–11,335)	9205 (6485–12,520)	7560 (5368–12,148)	.327
Neutrophil (/mm^3^)	3705 (2088–6118)	3550 (2048–5733)	3995 (2163–6620)	4130 (2283–5818)	.434
Lymphocyte (/mm^3^)	3560 (2228–5203)	3760 (2355–5208)	3495 (2293–5330)	2060 (1310–4103)	.015ᵃ
Monocyte (/mm^3^)	740 (510–1100)	800 (573–1120)	714 (500–1020)	510 (380–1003)	.025ᵇ
CRP (mg/L)	7.9 (1.0–26.0)	4.0 (0.0–17.5)	10.0 (2.0–30.8)	32.0 (6.5–52.0)	<.001ᶜ
NLR	1.1 (0.5–2.2)	1.0 (0.4–2.0)	1.1 (0.5–2.0)	2.7 (1.0–3.3)	.021ᵈ
MLR	0.22 (0.14–0.34)	0.23 (0.14–0.35)	0.22 (0.13–0.32)	0.26 (0.10–0.37)	.325

Values are median (IQR). CRP = C-reactive protein; NLR = neutrophil-to-lymphocyte ratio; MLR = monocyte-to-lymphocyte ratio. *p* values were calculated via the Kruskal–Wallis test; pairwise comparisons via the Dunn-Bonferroni post-hoc test. Institutional reference ranges: WBC 4,500–13,500/μL; neutrophils 1,800–7,700/μL; lymphocytes 1,000–4,800/μL; monocytes 300–900/μL; CRP < 5 mg/L; NLR 1.0–3.0; MLR 0.1–0.4. ᵃ Mild > Severe; Moderate > Severe. ᵇ Mild > Severe. ᶜ Severe > Mild; Moderate > Mild. ᵈ Severe > Mild

Multivariable binary logistic regression comparing severe versus non-severe outcomes (*n* = 322; 27 severe cases) identified underlying comorbidity as an independent risk factor for severe respiratory disease (OR 2.96, 95% CI 1.17–7.49, *p* = 0.022), along with CRP (OR 1.55 per 1 SD increase [≈32 mg/L], 95% CI 1.08–2.22, *p* = 0.018). Age < 1 year (OR 1.80, 95% CI 0.58–5.59, *p* = 0.308), age greater than 5 years (OR 1.99, 95% CI 0.76–5.17, *p* = 0.159), NLR, and monocyte count did not retain significance after adjustment (Table 5). The model AUC was 0.733.

**Table 5:** Multivariable binary logistic regression: risk factors for severe respiratory disease (Modified Tal Score ≥ 11 vs. < 11)

Variable	OR	95% CI	*p*
Age < 1 year (vs. 1–5 years)	1.80	0.58–5.59	.308
Age > 5 years (vs. 1–5 years)	1.99	0.76–5.17	.159
Any underlying comorbidity	2.96	1.17–7.49	.022
CRP (per 1 SD increase [≈32 mg/L])	1.55	1.08–2.22	.018
NLR (per 1 SD increase [≈2.9])	0.98	0.65–1.46	.903
Monocyte count (per 1 SD increase [≈490/μL])	0.76	0.47–1.22	.257

Analysis performed on *n* = 322 unique episodes with complete biomarker data (27 severe events). Reference category for age group: 1–5 years. Continuous predictors standardized prior to entry. Model AUC = 0.733

**Fig. 2:**
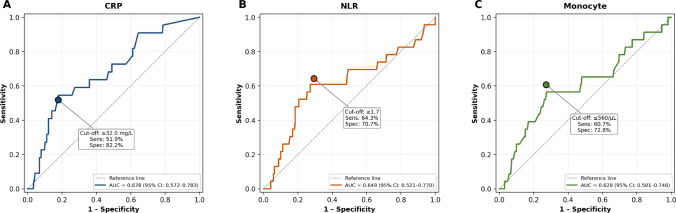
Receiver operating characteristic (ROC) curves for CRP, NLR, and monocyte count in predicting severe versus non-severe hMPV-associated LRTI at admission. Filled circles indicate optimal cut-off points determined by Youden’s Index. AUC values with 95% confidence intervals are displayed for each panel

ROC analysis identified optimal cut-off values for predicting severe respiratory disease: CRP ≥ 32.0 mg/L (AUC 0.678, 95% CI 0.572–0.783; sensitivity 51.9%, specificity 82.2%, negative likelihood ratio 0.59), monocyte count ≤ 560/μL (AUC 0.628, 95% CI 0.501–0.748; sensitivity 60.7%, specificity 72.8%, negative likelihood ratio 0.54), and NLR ≥ 1.7 (AUC 0.649, 95% CI 0.521–0.770; sensitivity 64.3%, specificity 70.7%, negative likelihood ratio 0.50) (Fig. 2). AUC values were modest (0.628–0.678), and negative likelihood ratios ranged from 0.50 to 0.59, indicating that values below these thresholds provided only moderate evidence against severe respiratory disease.

## Discussion

Of 421 cases with hMPV-associated LRTI, roughly half (47.0%) required hospitalization, and among the 198 hospitalized children, 28 (14.1%) developed severe respiratory disease. Comorbidity increased the risk of severe respiratory disease approximately threefold (OR 2.96, *p* = 0.022). CRP was the only inflammatory biomarker that retained significance after adjustment (OR 1.55 per SD, *p* = 0.018), though its diagnostic accuracy was modest (AUC 0.678) and a negative result could not reliably exclude severe respiratory disease (negative likelihood ratio 0.59). Neither age group nor other inflammatory indices (NLR, monocyte count) remained independently associated with severity, and viral co-detections had no significant relationship with clinical outcomes.

Our data support the established association between hMPV infection and increased severity in infants under 12 months [8, 9]. In univariate analysis, both infants and children older than 5 years showed higher proportions of severe respiratory disease. A similar age-related pattern has been documented in RSV infections [10], and a comparative study of RSV and hMPV reported that children hospitalized with hMPV tended to be older and carried a higher burden of comorbid conditions [11]. Overall, the findings show that younger, previously healthy children are at greater risk for severe RSV disease, while older children with underlying comorbidities represent the predominant high-risk population for severe hMPV-associated LRTI. In our cohort, 54.1% of children older than 5 years had at least one underlying comorbidity. The logistic regression model clarifies this overlap directly: after adjustment for comorbidity, age greater than 5 years was not an independent risk factor for severe respiratory disease (OR 1.99, *p* = 0.159), while comorbidity remained significant (OR 2.96, *p* = 0.022). This result supports the interpretation that the severity signal in older children reflects a progressive shift in the hospital-presenting population toward children with chronic conditions as age increases. On the other hand, the lower severity burden in the 1–5-year group likely reflects the predominantly healthy composition of this age cohort, as these children tend not to require hospital-level care for hMPV infection. Age < 1 year also did not retain significance in the adjusted model (OR 1.80, *p* = 0.308), which likely reflects limited statistical power given 27 severe events rather than a true absence of effect.

Our seasonal findings are consistent with recent epidemiological evidence that hMPV peaks predominantly during spring [12–14]. Spring and winter together accounted for 88.8% of all cases in our cohort. The association between severity and seasonality did not reach significance (*p* = 0.198), though both seasons concentrated the majority of severe cases. The suppression of hMPV circulation during the COVID-19 pandemic appears to represent a temporary epidemiological shift rather than a lasting change, with activity expected to rebound as public health restrictions ease [15]. Our cohort's observed pandemic-related decline and subsequent gradual rebound aligns with this expected trajectory.

Co-detections were common in our cohort (41.3%) but had no association with clinical severity. Because PCR identifies viral nucleic acid rather than replicating virus, detection does not confirm active infection; co-detected agents may represent residual shedding or incidental carriage rather than concurrent disease. This distinction is relevant when interpreting the absence of a severity effect: co-detection may overestimate true co-infection, diluting any real association. García et al. (2017) reported a similar 38% co-detection rate among pediatric hMPV cases [16]. In that study, adenovirus and rhinovirus were the most frequently detected pathogens, and the only significant difference between mono-detection and co-detection groups was a longer hospital stay. Zhao et al. (2022) reported a co-detection rate of 34.5% in a multicenter study, with rhinovirus as the predominant agent followed by RSV, adenovirus, and influenza A [17]. Rhinovirus was also the most common co-detected pathogen in our cohort, but the presence of concurrent viral agents did not correlate with severity.

The strong association between comorbidities and severe hMPV respiratory disease in our cohort is consistent with published evidence. Hospitalized children with hMPV frequently have chronic conditions such as asthma, prematurity, congenital heart disease, or malignancy [18]. Hibbert et al. (2025) reported that 41.2% of hMPV-positive children had comorbidities including prematurity, neurological disorders, or malignancies, and these were associated with prolonged hospitalization and higher rates of intensive care admission [19]. The comorbidity rate in our cohort (39.4%) is comparable and likely reflects the tertiary-care setting of our institution.

Yang et al. (2025) reported that among 131 children with hMPV-associated LRTI, 35.1% had severe respiratory disease, and these patients had higher neutrophil percentages and greater respiratory support requirements [18]. These findings parallel our own results. Published data on the relationship between hMPV severity and inflammatory ratios such as NLR and MLR remain limited. Guo et al. studied 357 patients with hMPV-associated LRTI and found that neutrophil counts, CRP levels, and NLR were elevated in the severe group (*n* = 102), consistent with our univariate findings [20]. That study did not report lymphocyte or monocyte counts. In our cohort, severe respiratory disease was associated with decreased lymphocyte and monocyte levels, which may reflect cellular consumption during acute inflammation. However, only CRP retained significance in multivariable analysis (OR 1.55 per SD, *p* = 0.018); the univariate associations of NLR and monocyte count were partially confounded by age and comorbidity status. ROC analysis confirmed that the diagnostic accuracy of all three biomarkers was modest (AUC 0.628–0.678). Negative likelihood ratios ranged from 0.50 to 0.59, well above the 0.1 threshold considered clinically useful for exclusion, indicating that values below the identified cut-offs cannot reliably rule out severe respiratory disease. These biomarkers may supplement clinical assessment but do not function as standalone triage tools.

## Limitations

The retrospective, single-center design may limit the generalizability of our findings. Severe respiratory disease accounted for only 6.7% (*n* = 28) of the cohort, which constrained the logistic regression model to six predictors and limits the statistical power of subgroup analyses. The findings should therefore be interpreted as associative rather than causative. NLR, monocyte count, and age groups did not retain significance after adjustment, and their univariate associations may be partially explained by confounding with comorbidity status. These results require validation in larger cohorts.

Modified Tal Score classifies severity based on respiratory parameters alone (respiratory rate, oxygen saturation, wheezing, and accessory muscle use) and does not incorporate systemic features such as fever, feeding difficulty, dehydration, or general clinical appearance. Children who were systemically unwell but had moderate respiratory scores would not be classified as severe under this instrument. Clinical decision-making in practice relies on a broader assessment, and our severity classification may therefore underestimate the true burden of clinically severe respiratory disease.

Our retrospective design and reliance on routinely collected registry data meant that detailed clinical information was not consistently available. ICD-10 subcategories of LRTI and treatment variables such as antibiotic regimens, intravenous fluid administration, and nutritional support could not be evaluated. Aggregate institutional data on total LRTI admissions and the broader distribution of respiratory viruses across the study period were also unavailable within the approved data extraction framework. True epidemiological prevalence of hMPV within the LRTI population could therefore not be calculated; all percentages reported in this study represent proportions of the study cohort.

Data on viral load and hMPV genetic subtypes were not available, which prevented evaluation of their contribution to disease severity. The Modified Tal Score was calculated retrospectively from medical records. Although all component parameters were documented at the time of presentation, this approach introduces measurement variability that prospective real-time scoring would avoid. Shifts in testing practices and healthcare-seeking behaviors during the COVID-19 pandemic may also have influenced the observed patterns of hMPV case distribution and severity.

## Conclusion

In this cohort of children with hMPV-associated LRTI, severe respiratory disease was infrequent (6.7%) and occurred predominantly in children with underlying comorbidities, which increased the odds approximately threefold (OR 2.96, *p* = 0.022). Age was not independently associated with severity after adjustment; the apparent severity signal in children older than 5 years reflected the higher comorbidity burden in that age group. Viral co-detections were frequent but unrelated to clinical severity. CRP retained an independent association with severe respiratory disease, but diagnostic accuracy for all three biomarkers was modest (AUC 0.628–0.678), and negative likelihood ratios (0.50–0.59) indicate that values below the identified thresholds cannot reliably exclude severe respiratory disease. Children with comorbidities warrant close monitoring regardless of age. Prospective multicenter studies incorporating viral load, genotype data, and standardized severity scores could validate these findings.

## Data Availability

No datasets were generated or analysed during the current study.

## List of Abbreviations

CRP: C-reactive protein
hMPV: Human metapneumovirus
LRTI: Lower respiratory tract infection
MLR: Monocyte-to-lymphocyte ratio
M-Tal: Modified Tal Score
NLR: Neutrophil-to-lymphocyte ratio
RSV: Respiratory syncytial virus
